# Implementation of EPR-Youth, a Client-Accessible and Multidisciplinary Health Record; A Mixed-Methods Process Evaluation

**DOI:** 10.5334/ijic.6905

**Published:** 2023-06-16

**Authors:** Janine Benjamins, Jan-Gerrit Duinkerken, Gerlinde den Hamer-Jordaan, Romay Canfijn, Rianne Koster, Emely de Vet, Annemien Haveman-Nies

**Affiliations:** 1 Icare JGZ, Blankenstein 550, 7943 PA, Meppel, the Netherlands; 2 Wageningen University and Research, Chairgroup Consumption and Healthy Lifestyles, Hollandseweg 1, 6707 KN, Wageningen, the Netherlands; 3 Stichting Jeugd Noord Veluwe, Stationsplein 18a, 8071 CH, Nunspeet, the Netherlands; 4 GGD NOG, Rijksstraatweg 65, 7231 AC, Warnsveld, the Netherlands

**Keywords:** Electronic Health Records, implementation, integrated care, child welfare, child health services

## Abstract

**Introduction:**

Client-accessible interdisciplinary health records potentially contribute to integrated care by facilitating collaboration and enhancing clients’ involvement in care. To achieve this, three Dutch organizations providing ‘care for youth’ developed a fully client-accessible electronic patient record (EPR-Youth).

**Objective:**

To evaluate the implementation of EPR-Youth and to determine barriers and facilitators.

**Methods:**

A mixed methods design combined system data, process observations, questionnaires and focus group interviews. Target groups were parents, adolescents, professionals using EPR-Youth, and implementation stakeholders.

**Findings:**

Client-portal acceptability was high among all clients. Client-portal adoption rate was high and differed between age groups and educational levels. Professionals’ doubts about acceptability, appropriateness and fidelity were partly due to lack of system knowledge. Implementation barriers were the complexity of co-creation, lack of clear leadership, and concerns about legal issues. Facilitators were clarifying vision and legal context, setting deadlines, and a pioneering spirit.

**Conclusion:**

The early implementation of EPR-Youth, the first Dutch client-accessible interdisciplinary electronic health record in ‘care for youth’ was successful. To enhance adoption among clients, group-specific barriers for portal-use should be determined. Professionals need additional training. Further research is needed to gain insight into client-portal access barriers. To benefit more from co-creation, an organizational change towards situational leadership is necessary.

## Introduction

During the last few decades, integrated care is worldwide considered a promising solution to reduce healthcare costs, improve patient experiences and enhance the quality of care [1]. Integrated care can be defined from different perspectives. For the purpose of this paper a health-system based definition is used, defining integrated care as person-centred care that is delivered in a way that ensures people receive a continuum of all possible different health services according to their needs throughout their life course [2]. Within this definition, person-centredness means that health systems and professionals consider individuals and families as participants in organizing care, appreciating a person’s health needs and expectations equally as the professional’s knowledge and expertise [3,4,5].

Integrated care can be supported on a functional level by the interdisciplinary use of electronic health records [6]. Research shows that shared use of electronic health records potentially contributes to interdisciplinary collaboration and to better quality of care [7,8]. Moreover, granting clients access to the contents of an electronic health records contributes to person-centred care because the transparency of a client-accessible health record improves communication between client and professionals and enhances a client’s involvement in their own care [9,10,11].

In the Netherlands, increasing costs and fragmented care in Youth Care have induced a necessary transformation towards integrated care since 2015. In that year, the responsibility for this transformation was transferred from the national to the local government. Six municipalities in the North-Veluwe region commissioned three local organizations to integrate their preventive and youth care services in centra for youth and family (CJG) and to develop a shared client-accessible electronic health record. With the implementation of this electronic health record, ‘EPR-Youth’, the CJG-organizations aimed for better interdisciplinary collaboration between CJG-professionals, for increasing client autonomy, and for improvement of perceived quality of care.

Developing an interdisciplinary client-accessible health record for care for youth, however, is a complex intervention facing specific challenges. Worldwide, development of client-accessible health records for adolescents has been hindered due to the complexity of confidentiality issues, and little research can be found on this topic [12,13,14]. In the Netherlands, EPR-Youth would be the first system for preventive child health care (PCH) to be fully transparent for both parents and adolescents and to be used interdisciplinary between preventive child health and youth social services. Furthermore, the virtual merging of three different organization to facilitate shared use of an electronic health record, requiring changes on organizational and individual professional level, enhances complexity.

The intended effects of EPR-Youth will only be achieved after successful development and implementation, meaning that the developed system supports the envisioned integrated working processes, and that professionals and clients use the new system in the way it was designed for. Therefore, an adequate evaluation of the development and implementation of EPR-Youth will add to the knowledge and understanding of delivering integrated care in the context of care for youth. The objective of this process evaluation is to investigate the development and implementation of EPR-Youth and to determine barriers and facilitators in the process.

## Methods

Reporting of the process evaluation was guided by the revised Criteria for Reporting the Development and Evaluation of Complex Interventions in healthcare (CReDECI2) guideline [15].

### Context

*Health system*: the Netherlands has established a high-quality preventive child health (PCH) system, underpinned by public health legislation. Over 90% of all Dutch children follow the full free program, consisting of 10 visits in the first year, 5 visits between 1 and 4 years and 5 visits between 4 and 18 years [16]. Historically, preventive healthcare for children aged 0–3 years was delivered by private organizations for medical homecare, whereas preventive healthcare for children aged 4–18 years was embedded in municipal health organizations and directed by local government. Local municipalities are financially responsible for the whole preventive child health program. Since 2015, with the introduction of the new Youth Act, responsibility for Youth Care, including youth social services, youth mental health and child protection, was transferred to municipalities as well.

**Figure 1:**
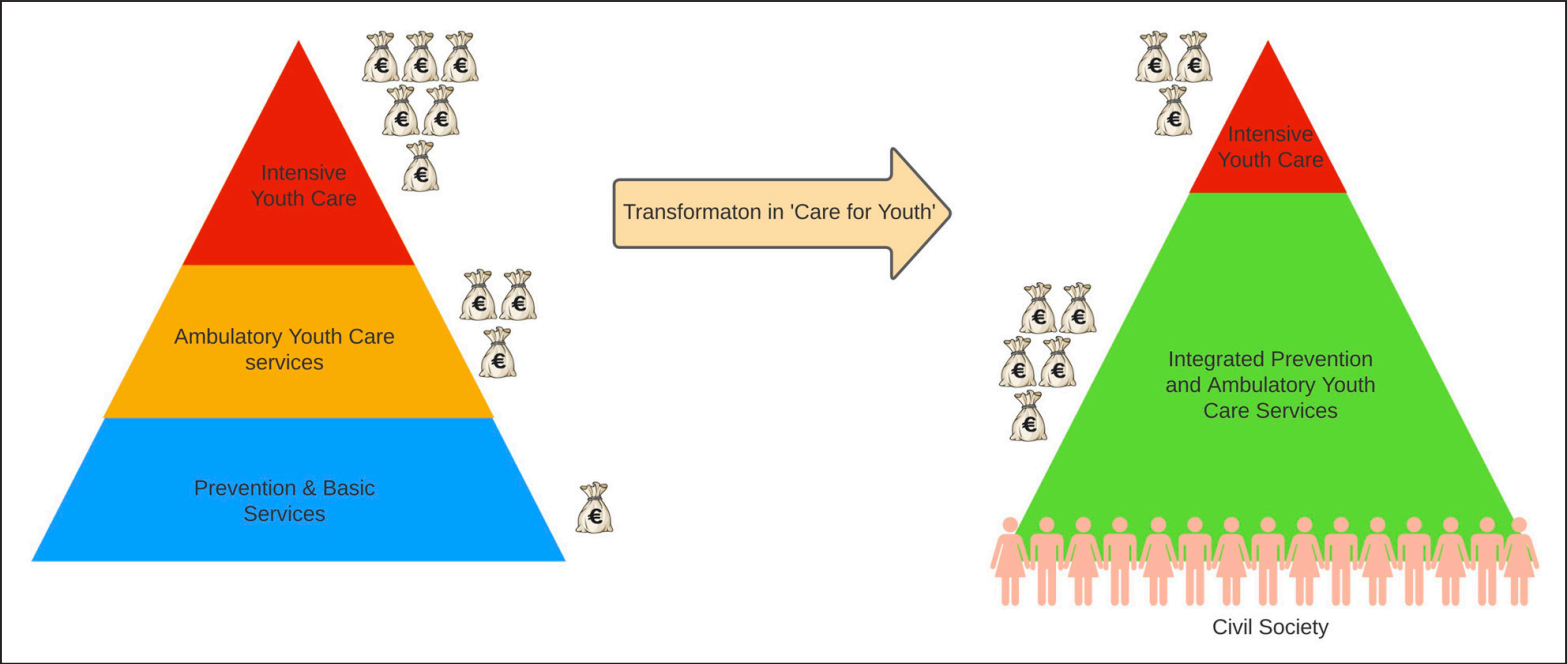
Transformation Youth Care in North-Veluwe region: make use of the strength of the ‘Civil Society, invest in prevention and integrate prevention and care to lower costs for intensive youth care (Benjamins et al, 2015).

*Transformation*: The transition of youth care to municipalities was accompanied by a challenge to reduce fragmentation of care within a lower budget. In the North-Veluwe region, six municipalities and three regional organizations for Preventive Child Healthcare and for Youth Care formulated a shared vision on transformation of care for youth, stating that integration of preventive services and youth care was needed, combined with a more client-centred attitude, to limit costs for care for youth (Figure 1) [17].

The three organizations should integrate their services, creating multidisciplinary teams in Centra for Youth and Family (CJG’s). Part of the integration assignment was the development of a multidisciplinary and fully client-accessible EPR-Youth, facilitating the client-centred approach.

*Legal aspects*: Due to privacy legislation, sharing electronic record between three different organizations was only possible after explicit and specific approval from a parent or adolescent. Dutch legislation provided adolescents with right of access to their record at the age of 12 [18]. Parental access was possible until their child was 16 years old, unless rejected by an adolescent from 12 years based on right to confidentiality. EPR-Youth anticipated on new legislation, expected in 2020, that would oblige healthcare organizations to provide patients with digital access to their health information [19].

### Description intervention

EPR-Youth was based on an existing preventive child health system, different from the systems previously used by the regional CJG-organizations. During the development phase, functionalities for youth care were added, as well as a client portal. Information from the existing electronic health records of the CJG-organizations was merged into EPR-Youth. All CJG-professionals, both from youth care and preventive healthcare, reported in EPR-Youth. The client portal provided parents and adolescents visiting the CJG, from now on referred to as ‘clients’, with full access to their own record. They would be able read all reports, add information, ask questions, and manage appointments.

Professionals were authorized to access a child’s record when they were involved with that child. Their access to a child’s record was visible in the client portal. Parents were given access to their child’s record until the age of 12. At the age of 12, the adolescent would get access, and parental access was prolonged only based on their child’s approval. Adolescent had the opportunity to keep specific information confidential between themselves and a professional.

The target population of this intervention consists of all 38,000 children aged 0–18 years who live in the region, their parents (52,800 persons) and all CJG-professionals.

### Implementation strategy

**Figure 2:**
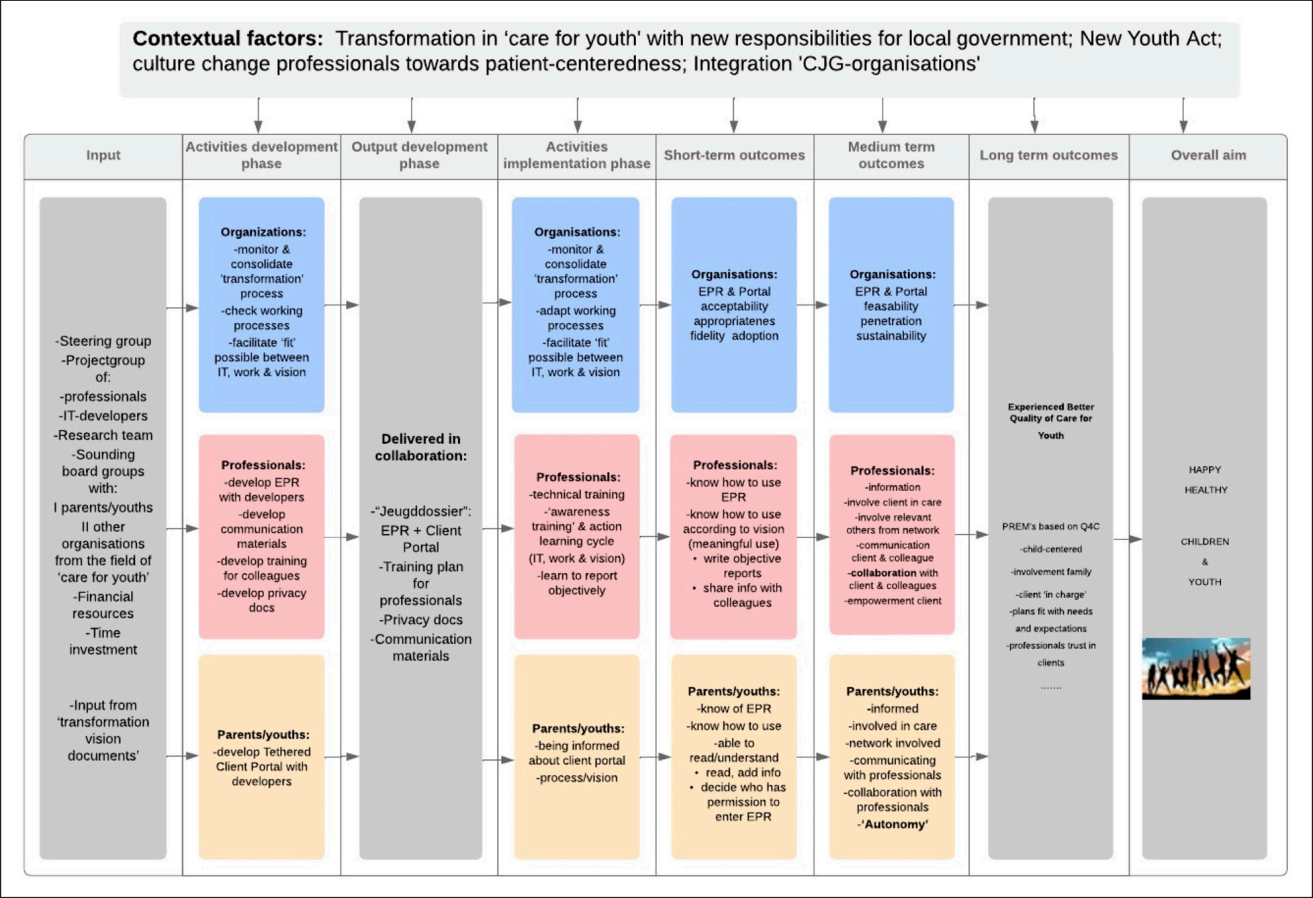
Logic model of change for EPR-Youth, based on the WK Kellogg Foundation logic model development guide.

The implementation of EPR-Youth was a complex intervention, encompassing more than the mere introduction of a new technological tool [20]. Use of EPR-Youth should also support the shared vision on integrated care and facilitate the necessary shift in professional behaviour. Furthermore, an organizational change was necessary, virtually merging working processes of three organizations. Based on system theory, stating that complex interventions will especially succeed if ideas and insights of all stakeholders are included, implementation of EPR-Youth was planned as co-creation between professionals, parents, adolescents, researchers, and IT-workers, including development as the first implementation phase [20,21]. The intended changes and deliveries, from development phase until long-term outcomes, are represented in a logic model of change (Figure 2), at an organizational, professional and client level [22].

A group of CJG-professionals, researchers, and IT builders, guided by a project leader, was appointed to develop and implement EPR-Youth together with parents and adolescents.

The project group was responsible for the process and were to deliver EPR-Youth, a training plan for professionals and a communication plan including communication materials; The project group reported to a steering committee, consisting of the managers of the CJG-organizations, a medical specialist, and the project leader. The steering committee monitored the process and only intervened when needed. A consultative group of parents and adolescents advised about layout and content of the client-portal.

### Evaluation

#### Process evaluation design

For this process evaluation, we used a mixed methods design, combining questionnaires, system data, focus group interviews, project documentation and observational reports. Data collection ran from May 2018 to November 2020 (see Table 1 for details). Following the theoretical framework for implementation research by Proctor et al, the early-stage implementation factors adoption, acceptability, appropriateness, and fidelity were chosen to be measured [23].

**Table 1:** Overview of chosen process indicators and implementation outcomes, corresponding data-collection method, examination period, and target group for the process evaluation of EPR-Youth.


INDICATOR/OUTCOME: *DESCRIPTION*	DATA	PERIOD	TARGET GROUP/ACTORS

**Delivery process:** *Has EPR-Youth been developed as intended and in accordance with the contract?*	Contract between IT-developer and CJG-organizations Document ‘system assessment’ Project documentation	Sept ’19 (immediately after implementation)	Not specified

**Implementation process and context:** *Analysis of legal/ethical, socio-cultural, geographical political and socio-economic contextual aspects that were affecting the implementation process*.	Verbatim transcripts project group meetings (34 × 3 hours), steering committee meetings (17 × 1,5 hours) and consultative group meetings (7 × 2 hours)	May ’18–Sept ‘19	Steering committee, project group, consultative group parents & adolescents

	Semi-structured focus group interviews with steering committee (n = 6) and project group (n = 8), 1,5 hours each.	June ‘20	Steering committee and project group

	Project documentation	Jan ’16–Sept ‘19	

**Acceptability:** *To what extent were users, both professionals and clients, satisfied with the intervention?* [23]	Professionals’ questionnaire (n = 66): *experienced ease-of-use and experienced usefulness*	Feb ’20	CJG-professionals

	Questionnaires parents (n = 914) and adolescents (n = 89): *desirability client-access, actual access, and experienced ease-of-use*.	Sept–Nov ‘20	Parents and adolescents that visit a CJG

	Semi-structured focus group interviews with professionals (n = 12), parents (n = 8) and adolescents (n = 4): *how do professionals and clients experience the use of EPR- Youth and the client-portal?*	Nov ‘20	Professionals and clients

**Adoption:** *To what extent were clients using the client-portal?* [23]	System data: *monthly and total number of clients that logged on to the portal*	Sept ’19–Dec ‘20	Clients

	Questionnaires parents (n = 914) and adolescents (n = 89): *percentage of respondents that logged on to the client-portal*	Sept–Nov ‘20	Parents and adolescents that visit a CJG

**Appropriateness:** *To what extent does EPR-Youth match with working processes of professionals?* [23]	Meeting reports of project group and steering committee	May ’18–Sept ‘19	Steering committee and project group

	Semi-structured focus group interviews with professionals (n = 12): *Do professionals feel a match between EPR-Youth and* *their working processes?*	Nov ‘20	CJG-professionals

**Fidelity:** *To what extent are professionals and clients using EPR-Youth and the client-portal as intended, in accordance with the vision on transformation?* [23]	Semi-structured focus group interviews with steering committee (n = 6) and project group (n = 8), 1,5 hours each.	June ‘20	Steering committee and project group

	Semi-structured focus group interviews (1,5 hours) with professionals (n = 12), parents (n = 8) and adolescents (n = 4): *Do participants experience that EPR-Youth is supporting professionals to work in accordance with the vision on transformation?*	Nov ‘20	Professionals and clients


#### Target groups and recruitment

The research population consisted of different target groups: parents, adolescents, and professionals that were using EPR-Youth; members of the steering committee, guiding the development and implementation; professionals co-creating EPR-Youth with IT-developers. Table 1 describes which target group was approached in which part of the study. During the development and implementation phase, observational reports were made for every project meeting. One year after introduction of EPR-Youth, all clients visiting a CJG received the clients’ questionnaire. From the clients that indicated willingness to participate in a focus group, two focus groups were selected by purposive sampling, including clients from all six municipalities, both parents and adolescents, both male and female, visitors of different CJG-services, and representing different educational levels.

The professionals’ questionnaire was distributed among 92 CJG-professionals from all three organizations, representing all available disciplines. For the focus group interviews with professional users and with the project group, purposive sampling was used to ensure that participants represented all disciplines and organizations involved, both sexes and different levels of working experience. In the focus group interview with the steering committee, all members of the steering committee were included. One member was absent and was interviewed separately. Appendix 1 provides an overview of characteristics of all focus group participants.

#### Measurements

##### Fidelity of delivery process

To evaluate whether EPR-Youth was delivered as intended, a system assessment was performed by the first author (JB) and members of the project group, comparing EPR-Youths’ delivered functionalities and actual timeline of delivery with project documentation and with the project contract.

##### Implementation process

The first author (JB) participated as participating observer in all steering committee meetings, all project group meetings and all consultative group meetings with parents and adolescents. All meetings were audio-recorded. Project documentation was used for triangulation. The focus groups with project group members and with the steering groups were used to further define barriers and facilitators in the implementation process.

##### Implementation outcomes

Acceptability, adoption, appropriateness, and fidelity were chosen as implementation outcomes.

*Acceptability*, meaning ‘the perception among implementation stakeholders that a given innovation is agreeable or satisfactory’ [23] was assessed among both professionals and clients with questionnaires. Based on the Technology Acceptance Model, the professionals’ questionnaire contained questions about ‘perceived ease-of-use’ from the System Usability Scale (N = 9, Cronbach’s alpha = 0.92) and questions about ‘perceived usefulness’ from a questionnaire by Davis et al. (N = 4, Cronbach’s alpha = 0.84) [24,25]. The clients’ questionnaire was embedded into client satisfaction survey, that was administered to all clients who visited a CJG. The questionnaire contained three questions about ‘ease-of-use’, two about ‘adoption’, one about ‘desirability of a client accessible EPR’. In both questionnaires, respondents were asked to rate their answers on a 5-point Likert scale, ranging from ‘totally agree’ to ‘totally disagree’ (Appendix 2). To match high scores with a positive opinion, all scores were reversed, except for four questions in the professionals’ questionnaire, that were reversely worded (Figure 4). Results were presented in a descriptive way.

*Adoption*, meaning ‘intention, initial decision, or action to try or employ an innovation’ [23], was assessed on client level using system data. The number of clients that logged on were counted and presented per month and as a total. To calculate number of log-ons per client, multiple log-ons on the same day were counted as one.

Focus groups were conducted with clients and professionals to assess *appropriateness*, meaning ‘fit with working processes’, and *fidelity*, meaning ‘being used as intended’ and to deepen understanding of *acceptability* and *adoption* [23].

#### Data-analysis

Quantitative data were analyzed using IBM SPSS Statistics 25 and Microsoft Excel. To test for differences *in adoption rate based on demographic characteristics*, demographic data of portal-using clients were compared with those of the source population using Chi-square tests. Client-portal access percentages, as reported in the questionnaire, were tested for difference according to sex, age or native country with Chi-square or Fisher’s exact tests. Client user experience scores were tested for differences according to educational level and native country. Kruskal-Wallis was used as omnibus test and the Mann Whitney-U as post-hoc test. For professionals’ user experiences, two dimensions were defined: perceived ease-of-use and perceived usefulness. For each dimension, an average score was calculated. These scores were tested for differences according to age and organization, using one way ANOVA as omnibus test, and Tukey HSD as post-hoc test.

All project meetings, focus group interviews and individual interviews were audio-recorded, transcribed verbatim, and analysed in ATLAS.ti, version 8 and 9. Four researchers working in pairs (RK, JB, RC, GJ) performed a thematic analysis, starting from the themes ‘merging three systems’, ‘client-access’ and ‘general project process’. Based on relationships and cohesion between initial codes, a codetree was built with five main themes (Appendix 3), adding ‘vision’ and ‘bottlenecks, benefits and yield’ as new emerging themes. Finally, interpretation of the themes was discussed with all authors.

## Findings

### Fidelity of delivery process

**Figure 3:**
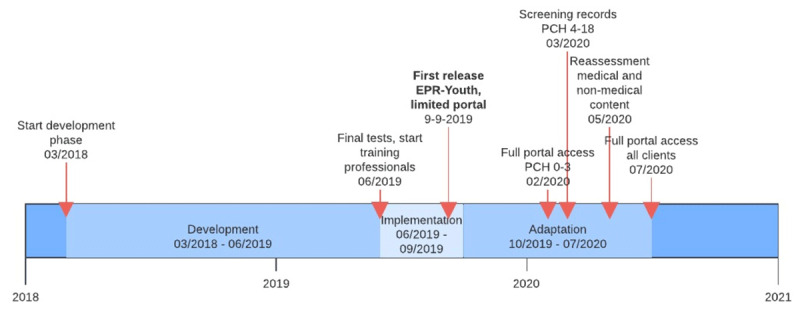
Timeline for development and implementation of EPR-Youth.

After a development phase of 18 months, EPR-Youth was introduced in September ’19, which was six months later than intended. The system could be used by all CJG-professionals. Some adjustments were made during the delivery process (Figure 3).

First, the client-portal started with limited functionalities: clients could manage appointments, ask questions, and had access to vaccination status and growth data. Full access to visit notes was postponed until February ‘20 because the client consultation group had expressed safety worries during the final tests.

Second, all clients were to be informed personally about the client portal. However, the planned mailing to adolescents and parents of school-aged children was cancelled because the start of the COVID-19 pandemic required full attention of the municipal health organization.

Third, portal access was limited to parents of pre-school children at first because preventive health professionals working with school-age children and adolescents wanted to screen all health records, ensuring nothing was reported about third parties, before sharing the content with their clients. They finished the screening process in June ’20.

Finally, the strict division between medical content, visible only for medical professionals, and non-medical content, visible for all CJG-professionals, was reassessed in May ’20. The reason for this reassessment was that the strict division prohibited youth care workers to see all relevant information about children’s health, which hindered adequate delivery of care.

When these changes in the delivery process were completed, most members of the steering committee and project group considered the implementation of EPR-Youth successful, because adequate interdisciplinary use and full portal access were possible by now. Simultaneously, there was still room for improvement.

### Barriers and facilitators in implementation process

From the qualitative analysis of focus group interviews and project meetings, the following themes emerged as barriers: ‘complexity of co-creation’, ‘lack of leadership’, ‘concern about legal aspects’ and ‘lack of communication’, whereas ‘structuring the process’, ‘clarifying the vision’, ‘pioneering spirit’ and ‘resolving legal issues’ were defined as facilitator. Themes and significant quotes are shown in Table 2.

**Table 2:** Summary of qualitative themes and sample quotations.


THEME	RESPONDENT	SETTING	QUOTE

***A1: Implementation process, barriers***			

Complexity of co-creation	Project leader	Project group meeting	*We are going to use one EPR-system with three organizations, meaning we’re going to merge virtually. I think we underestimated the complexity of that*.

	Preventive health worker	Focus group project members	*We have had a great deal of trouble pretending to be one organization, while we are not at all…. We are not working from one vision*.

Lack of leadership	IT worker	Focus group project members	*A plan of action was lacking, as well as clarity about everyone’s role*.

	Manager	Focus group steering committee	*The steering committee, including myself, too easily assumed that everyone knew the ultimate objective*.

	Preventive health worker	Focus group project members	*The project leader kept saying that we had to ask the steering committee about issues*.

Concerns about legal aspects	Father	Consultative group of clients	*As a parent, I really don’t want that my child’s medical letters can be downloaded to my computer without warning. That feels unsafe*.

	Preventive health worker	Project group meeting	*Professionals must discuss with an adolescent whether their parents are allowed to access their record, but they must speak with the parents as well*.

	Youth care worker	Project group meeting	*Maybe more medical information should be shown in the general record because some of that is really relevant for youth care workers*.

	Adolescent	Focus group clients	*As a child, especially when you grow up, you want to decide who can access your record and who can read it*.

Lack of information	Preventive health worker	Focus group professionals	*A year ago, information has been added to the invitation letter parents of newborns received, but we did not inform parents of older children*.

	Mother	Focus group clients	*I think you should really explain the client portal… to every parent. Don’t assume that they will find the client portal by themselves*.

***A2: Implementation process, facilitators***			

Structuring the process	Youth care worker	Focus group project members	*The deadline when EPR-Youth was delivered had to be met at all costs. Because the deadline was clear we were able to make rigorous decisions*.

	Preventive health worker	Focus group project members	*This is what people had been suggesting for so long: one group should be working on the content, and another on the newsletter, the user’s manual etc*

Clarifying the vision	Preventive health worker	Focus group project members	*Once everyone clearly understood the ‘why’, decisions were quickly made*.

	Medical specialist	Focus group steering committee	*Now that professionals begin to understand the vision, the resistance among them has considerably decreased*.

Pioneering spirit	Youth care worker	Focus group project members	*Having taken up this challenge together that no one had tackled before made the difficult process more tolerable*.

	Youth care worker	Focus group project members	*It’s nice to tell that this project is unique in the Netherlands*.

Legal aspects	Medical specialist	Project group meeting	*From July 2020, everybody will be legally obliged to provide digital access to health records*.

	Project leader	Project group meeting	*In my opinion a thorough legal assessment framework has been built already, and a legal guide has been provided*.

***B: Implementation outcomes***			

Acceptability	IT-worker	Focus group project members	*We have had relatively few start-up problems. Professionals ask practical questions about the record, but nothing like “what a miserable system” or “we can’t work with this”*

	Preventive health worker	Focus group professionals	*I really like this system, EPR-Youth is easy to fill in*

	Adolescent	Focus group clients	*When you enter the website you need to verify in four steps. It really takes time before you are able to read things*.

Adoption	Adolescent	Focus group clients	*Somebody asked if we wanted to complete a questionnaire and then we read about EPR-Youth. Otherwise, we would not have known*.

Appropriateness	Youth care worker	Focus group professionals	*As a youth care professional, it feels as if I am a visitor that is ‘allowed’ to report in a preventive healthcare record*.

	Youth care worker	Focus group professionals	*Sometimes I think how I am going to report all this, because I feel a 12-year-old should not have to read that*.

Fidelity	Preventive health worker	Focus group professionals	*When a child starts at school, you don’t have to transfer the record to the school doctor, because they continue working in the same system*.

	Manager	Focus group steering committee	*With EPR-Youth it is no longer possible to report things that clients are unaware of*.

	Youth care worker	Focus group professionals	*I have a lot of colleagues who still write their referrals in MS Word*.


#### Barriers

Complexity of co-creation: The project proceeded slower than expected, partly due to the complexity of the co-creative change process. Underlying to this complexity were differences between organizations. Whereas the members of the steering committee shared one vision about care for youth, this vision appeared not to be embraced throughout all three organizations, causing recurring substantive and legal discussions and interpersonal tensions in the project group.

Lack of leadership: The project group members felt the project was lacking a plan with clear division of responsibilities. Although the steering committee expected the project group to take ownership over the process, the group had not experienced ownership at the start, nor had the project leader required ownership from them. Maintaining balance between self-organizing as a project group and receiving directions from the steering committee was a recurring theme, both within the project group as between project leader and steering committee. Whereas the project leader urged the steering committee to influence professionals’ attitude and behaviour, they held back instead, emphasizing that change of attitude was a change process that would happen gradually over time. Members of the steering group acknowledged that their reluctance to take leadership might have caused the project group to feel lost sometimes and that they could have intervened earlier, especially in recurring discussions about vision.

Concern about legal aspects and privacy: During the development phase, both professionals and clients expressed their concerns about legal issues and about privacy. Clients expressed worries about safety of the client portal, eventually leading to postponement of full opening of the client portal (Figure 3). Professionals were concerned if EPR-Youth was being developed in accordance with new privacy legislation when professionals of three different organizations were allowed to work in the system. Furthermore, professionals had doubts about sharing sensitive information with young adolescents and were unsure how to deal with parents’ confidentiality rights in difficult situations, e.g. divorce, child abuse.

#### Facilitators

Structuring the process: Both the project group and the steering committee acknowledged the benefit of setting strict deadlines. This initiated a division of the project group in smaller task groups, which had proven very helpful and had contributed to the sense of ownership.

Clarifying the vision: The project group valued the process of clarifying the vision, although it was a time-consuming process. They felt this process had eventually enabled them to come to clear decisions. Moreover, the steering committee reported that the process of jointly clarifying the vision had reduced resistance.

Pioneering spirit: Everybody was aware of the pioneering character of this project, realizing that there was no earlier example that could be copied. This awareness created a feeling of pride, both in the steering committee and in the project group, and a strong will to complete the project successfully.

Legal aspects: To manage the doubts that professionals expressed about legal issues, a legal expert was consulted and a ‘frequently asked questions’ document was written, serving as a guide for both professionals and clients. Furthermore, the steering committee emphasized that the oncoming legislation, obliging every healthcare organization to digitally share record contents with their clients, contributed to the acceptation of EPR-Youth.

### Implementation Outcomes

#### Acceptability

**Figure 4:**
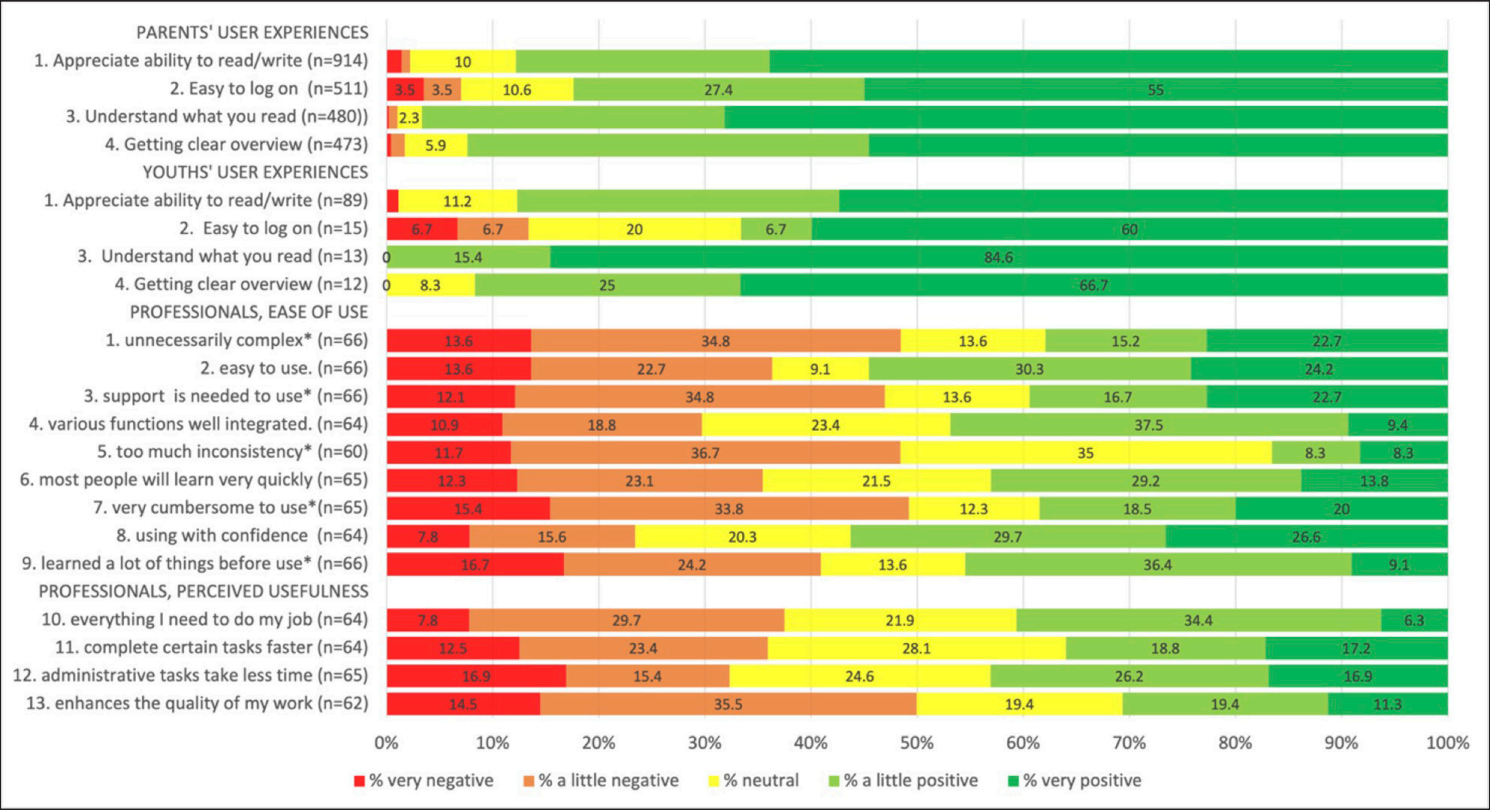
Clients’ and professionals’ user experiences with EPR-Youth. Respondents scored on a 5-point Likert scale, ranging from ‘absolutely’ (1) to ‘not at all’ (5) for clients, and ranging from ‘totally agree (1)’ to ‘totally disagree’ (5) for professionals. Scores were reversed for all questions, except the ones marked with an asterisk, resulting in low scores representing a negative opinion and high scores representing a positive opinion. In the client questionnaire, Q2–4 were answered only by parents and adolescents that had accessed the client-portal.

From the 911 parents and 87 adolescents that completed the clients’ questionnaire, 490 parents and 14 adolescents reported they had logged on to the client-portal and responded on the user experience questions (Figure 4). Clients were predominantly positive about easy access, comprehensibility, and clear overview. No difference in scores were found according to educational level or native country.

The professionals’ questionnaire was completed by 66 of the 92 (72%) invited CJG professionals (Figure 4). A statistically significant difference for ease-of-use was found between different organizations, and between age groups. Professionals delivering preventive healthcare to pre-school children experienced EPR-Youth easier-to-use (Mean = 3.4, SD = 1.0) than youth care professionals (Mean = 2.6, SD = 0.9), as determined by Tukey HSD post-hoc test (p < 0.001). Professionals aged 60 years and older experienced EPR-Youth easier-to-use than professionals between 40 and 50 years of age (Mean = 3.8, SD = 0.7 vs Mean = 2.4, SD = 0.9; p = 0.02).

In the focus groups, clients mainly reported that the client-portal was easy to use although adolescents considered the obligatory two-factor authentication time-consuming. Professionals differed in their opinion about ease-of-use, varying from ‘easy to fill in’ to ‘a nuisance’. They particularly appreciated new time-saving functionalities, like automatically generating referrals or indication statements. However, many professionals appeared not to be familiar with all possibilities of EPR-Youth.

#### Adoption

System data showed that 5174 clients had logged in to the portal in the period September 2019 until December 2020.

In the first five months, the monthly number of portal users slowly increased, then stabilized around 1200 portal users monthly. Table 3 shows that most portal users were women (87.1%), and most users were aged between 30 and 44 years (76.6%). Compared to the average local population, few portal users were of non-Dutch nativity. The average number of logons per person was 3.68 (Median = 2.0; SD = 4.1) in 15 months, ranging from 1 to 47 logons. No differences were found in logon frequency, according to sex, age, and native country.

**Table 3:** Demographic characteristics of portal users (from system data), compared with general population of the North Veluwe region, aged 15 to 65. Source: Dutch Central Bureau for Statistics.


TOTAL		NUMBER OF USERS (PERCENTAGE) 5174	PERCENTAGE AMONG INHABITANTS NORTH- VELUWE, AGED 15–65 YEARS

Sex^a^	Male	659 (12.7)	50.5

	Female	4509 (87.1)	49.5

	Unknown	6 (0.1)	

Age^b^	15–29 years	1107 (21.4)	28.4

	30–44 years	3964 (76.6)	27.6

	45–64 years	102 (2.0)	44.0

Native country^c^	Netherlands	4895 (94.6)	92.4

	Surinam	5 (0.1)	0.2

	Netherlands Antilles	5 (0.1)	0.1

	Turkey	28 (0.5)	1.0

	Morocco	15 (0.3)	0.5

	Europe, North America, Oceania, Indonesia, Japan	105 (2.0)	2.8

	Other country	121 (2.3)	3.1


^a^ Significantly different from North Veluwe population (Chi^2^-test (1) 2945.942, 2-sided p < 0.001). ^b^ Significantly different from North Veluwe population (Chi^2^-test (2) 6671.353, 2-sided p < 0.001). ^c^ Significantly different from North Veluwe population (Chi^2^-test (6) 41.644, 2-sided p < 0.001).

The clients’ questionnaire showed that logon percentages among parents differed, according to educational level, family composition and children’s age (Table 4). These differences did not appear among adolescents. Parents of children aged 0–3 years, reported most often that they had logged on to the client-portal. As reasons to log on to the portal, clients mentioned: checking or managing appointments (72.4%), reading what was discussed (54.3%), asking a question (16.7%) and adding or changing information (2.5%). In the client focus groups, most participants reported they had not been aware of the existence of the client-portal until they were invited to complete the clients’ questionnaire.

**Table 4:** Comparison of login percentages (between brackets) among respondents of the client questionnaire, according to socio-demographic characteristics.


	PARENTS (n = 911)						ADOLESCENTS (n = 87)					
	
	LOGGED IN n = 490 (53.8%)		NO PORTAL USE n = 421 (46.2%)		STATISTIC	2-SIDED p-VALUE	LOGGED IN n = 14 (16.1%)		NO PORTAL USE n = 73 (83.9%)		STATISTIC	2-SIDED p-VALUE

**Educational level**												

Low	32	(50.8)	31	(49.2)	χ^2^ = (2) 9.285	0.01	6	(15.0)	34	(85.0)	χ^2^ = (1) 0.671	0.41
	
Middle	207	(50)	207	(50)			NA		NA			
	
High	230	(60.5)	150	(39.5)			7	(22.6)	24	(77.4)		
	
Missing	21		33				1		15			

**Family composition**												

2-Parent family	452	(58.9)	284	(41.1)	χ^2^ = (1) 49.523	<0.001	5	(16.1)	26	(83.9)	Fisher’s exact	<0.001
	
Other situation	16	(18.8)	69	(81.2)			7	(15.9)	37	(84.1)		
	
Missing	22		36				2		10			

**Native country**												

Netherlands	431	(54.5)	360	(45.5)	χ^2^ = (1) 0.447	0.504	12	(17.4)	57	(82.6)	Fisher’s exact	0.58
	
Other country	15	(48.4)	16	(51.6)			0	(0.0)	6	(100.0)		
	
Missing	44		45				2		10			

**Age children**												

0–3 y	401	(77.0)	120	(23.0)	χ^2^ = (1) 264.065	<0.001	NA					
	
4–18 y	67	(20.2)	265	(79.8)			NA					
	
Missing	22		36				NA					


#### Appropriateness

When launched in 2019, EPR-Youth supported the most important working processes of CJG professionals. In the professional focus groups, however, preventive health care workers and youth care workers reported different opinions. Whereas preventive health care workers felt that EPR-Youth supported their working processes better than their old system, youth care workers missed a good match with their working processes and sometimes felt as if they were ‘visiting’ someone else’s system. Some professionals experienced difficulties reporting complex family situations, now that adolescents aged 12 and older had full access to their own health record. Before introduction of EPR-Youth, sensitive information considering parents, for instance during a divorce, could be registered without children reading the information. In EPR-Youth, however, information could not be shielded from the adolescents. Consequently, professionals faced difficult decisions what was relevant to report and how to report it.

#### Fidelity

To investigate implementation fidelity, we discussed with professionals in the focus groups whether the system was being used and implemented as intended. Professionals reported that EPR-Youth facilitated interdisciplinary collaboration, and that the ability to read record content and to plan appointments enhanced parent’s involvement in care. One member of the steering committee considered transparency to be the most important achievement because it was no longer possible to report about clients without their knowledge.

Nevertheless, the system was not fully used as intended. For instance, the possibility to manage appointments was offered by only one CJG-organization. Furthermore, some professionals appeared to lack knowledge of functionalities and struggled to make EPR-Youth work for them, concordant with the regional vision. To increase implementation fidelity, professionals requested additional training.

## Discussion

The aim of this comprehensive process evaluation was to investigate the implementation of EPR-Youth and to determine barriers and facilitators. With the implementation of a fully client-accessible health record that facilitated the working processes for three different organizations a strong basis has been created to deliver integrated care. However, client portal adoption differed between subgroups, as did acceptability, appropriateness, and fidelity among professionals. ‘Complexity of co-creation’, ‘lack of leadership’, ‘concern about legal aspects’ and ‘lack of communication’ proved to be barriers in the implementation process, whereas ‘structuring the process’, ‘clarifying the vision’, ‘a pioneering spirit’ and ‘resolving legal issues’ proved to be facilitators.

### Barriers and facilitators in the implementation process

Complexity of collaboration and lack of leadership were experienced as the most important barriers during implementation. These barriers can be interpreted as a side-effect of the choice to develop and implement EPR-Youth in a step-by-step co-creational process with relevant stakeholders [20,26,27]. Co-creation is a non-linear process, which is very suitable for innovation [28]. In this process, participants must share opinions, acknowledge divergent perceptions, challenge assumptions, and finally work through disagreements [29,30,31]. The members of our project group, coming from three different organizational cultures, discovered they had to go through this time-consuming process to reach the point where they could begin to search for inclusive solutions and come to a deepening sense of connection with each other [30,31,32,33].

The disruptive character of co-creational processes requires situational leadership that is adapting guidance of the participants to each phase of the process [34,35]. At the start, the group is in search for direction and objectives. Therefore, leadership needs to be directive, setting clear goals [29,35]. The discussion and polarization phase requires a coaching leadership style, keeping the group focused on the vision, stimulating the search for joint solutions and creating space and time for renewal processes [36]. In the last phase of co-creation, responsibility for shared goals will develop within the group, requiring more facilitative instead of directive leadership [29,35]. The steering committee chose a facilitative leadership style from the start, immediately expecting shared ownership of goals in the project group. Eventually, shared interpretations were formulated, and the feeling of solidarity and commitment within the project group increased. However, situational leadership, adapting to the changing needs and managing the participants’ expectations, could have diminished the turbulence of the project.

The awareness of joint commitment to the creation of something new, labelled as ‘sense of pioneering’, was considered an important ingredient for successful innovation. Senge et al wrote about the importance of joint commitment: “virtually every significant change initiative that we have seen starts with a genuine partnership among a small number of deeply committed individuals” [37]. In the context of change management, Clemmer and Warrick use the term ‘change champions’: individuals in various segments of the organization who make indispensable contributions to initiating, facilitating and implementing change [38,39]. Although they did not sense ownership at first, project group members implementing EPR-Youth eventually developed into ‘change champions’, initiating and supporting the intended change among professionals and clients using EPR-Youth.

Concerns about legal issues and privacy proved a barrier as well, and not only in this project.

Development of patient-accessible records for adolescents is hindered worldwide by the struggle of guarding adolescent’s rights to confidentiality [12,13,14]. However, we encountered another dilemma, representing the opposite side of the same coin, when professionals expressed concerns about possible violation of parent’s privacy due to adolescent record access. The dilemma is caused by a conflict between the adolescents’ right of access to their own health record, and the parents’ right to confidentiality over their personal information [14]. A child’s health record contains more information than just about the child’s health. Professionals in child and adolescent care, viewing from a biopsychosocial perspective, gather information about family circumstances and parent’s health issues as well and report those in the child’s record [40,41]. Professionals in our focus groups expressed worries that possibly stigmatizing information about parents would be disclosed that might be harmful for a 12-years-old to read. Literature pays little attention to protection of parent’s privacy in relation with client-accessible records for child and adolescent healthcare. The topic, however, certainly needs more attention as Bayer et al rightfully state [14].

### Implementation Outcomes

Self-reported client portal adoption was 16.1% among adolescents. Among parents, portal adoption was 53.8%, which is twice as high as internationally reported portal adoption rates in healthcare [42]. However, major differences in adoption rate were reported between parents of pre-school children and parents of adolescents and school-aged children. One explanation might be that parents of pre-school children had been informed individually by email, whereas the planned mailing by post to adolescent and parents of older children was cancelled due to COVID-19. Another possible explanation was the higher frequency of preventive check-ups in the pre-school period. Finally, parents of pre-school children were more inclined to access their client-portal because they were offered the opportunity to manage their appointments in the portal, whereas the municipal health service, delivering preventive child healthcare to school-aged children and adolescents and their parents, only offered the option to view appointments.

Client-portal adoption was highest among high-educated and native Dutch clients, which was in line with previous research [43,44,45,46]. Unexpectedly, experienced ease-of-use was not related to educational level or native country, which we considered hopeful. Nevertheless, further research will be needed to gain insight in other barriers for portal access, because lower portal adoption rates among lower educated groups and clients of non-Dutch nativity could be a sign of ‘digital divide’, with the possible risk of enhancing socio-economic health differences [47].

Professionals differed in opinion about acceptability, appropriateness, and fidelity of EPR-Youth. Their lack of knowledge of all system functionalities could be explained by the natural differences in any innovation process between early adopters and so-called ‘laggards’ [48]. However, professionals also experienced some mismatches between working processes and EPR-Youth, cherishing old habits and refusing to adopt new working methods that were supported by EPR-Youth. These issues go deeper and need to be understood from the nature of complex interventions, generating change on the level of technology, organization, and people (professionals and clients) [21]. Co-creating complex interventions requires interaction over time between the technological, institutional, and social components and their context, in a continuous cycle of feedback and learning [21,49]. Implementing EPR-Youth, the technological development continued as planned, while the changes in professional attitude and adoption of newer working processes were delayed due to the COVID-19 epidemic which put the action-learning program on hold.

In this light, ‘having developed a client-accessible system all professionals can work with’ can be considered a success on technology level. Simultaneously, to complete the implementation on organizational, professional and client level, it is important to continue the cycle of learning and feedback [36]. Part of the solution on the ‘people’ level could be the requested training for professionals. On organizational level, a structural change towards situational leadership, knowing when to be directive and when to guide with room for renewal, is important.

### Strengths and limitations

The combination of both quantitative and qualitative data from different sources, e.g. system data, project documentation, observation reports, questionnaires and focus group interviews allowed us to view the results from all relevant perspectives.

However, distributing the professional questionnaire only 5 months after introduction of EPR-Youth might have influenced the results for ‘acceptability’ in a negative direction, because professionals still reported insufficient familiarity with all system functionalities. Moreover, including only CJG-colleagues who were already in service when EPR-Youth was introduced, left out possible positive opinions from newer colleagues. In focus groups, however, newer colleagues were included, adding their more positive opinions to the overall picture of the system’s easiness-of-use.

JB’s active participation in meetings of project group, steering committee and client consultative group can be considered both a strength and a limitation [50]. From an empirical positivist perspective, interference with the process is unwelcome, because generalizability of outcomes will diminish and the researchers’ objectivity could decline [51]. In action research, however, knowledge is produced through interaction with the process and its participants [52]. Through continuous reflection on the process, delivering feedback to the steering committee and project group, JB contributed to the achievement of project goals [51,52]. The following measures were taken, aiming for intersubjectivity: focus group interviews by an independent reviewer; co-analyzing with researchers not involved in the implementation process; member check on both interview transcripts and all quotes in this paper [53].

The earlier mentioned COVID-19 pandemic also influenced the data collection. Professionals’ questionnaires were completed just before the pandemic, whereas client questionnaires that were planned one month later had to be postponed six months. Therefore, the implementation period had been shorter for professionals. That might have caused a lower perception of ease-of-use, due to lack of familiarity with all functionalities, whereas clients reported more positively because they had had more time to adjust.

## Conclusion

The first implementation stage of EPR-Youth, the first Dutch client-accessible health record that facilitates both preventive child health and youth care, was successful. However, more time and effort are needed to complete implementation on organizational and personal level. To inform clients about the existence of EPR-Youth, more communication is needed, especially towards groups with lower adoption rate. Further research is needed to gain insight into barriers for client-portal access. To enhance acceptability among professionals, and a better understanding of the match between EPR-Youth, working processes, and organizational vision on care for youth, we recommend additional training.

Although co-creation was an essential ingredient to reach project goals, situational leadership with more direction at the start and room for disruption is needed to guide the process.

## Data Accessibility Statement

The datasets generated during and/or analysed during the current study are deposited in the DANS EASY repository, DOI: https://doi.org/10.17026/dans-znd-cgwm, and will be available after an embargo period of one year. Until then, the datasets can be retrieved upon request from the corresponding author.

## Additional Files

The additional files for this article can be found as follows:

10.5334/ijic.6905.s1 Appendix 1. Overview of characteristics of focus group participants, shown separately for steering committee (n = 6), project group (n = 8), clients (n = 12), and professional users (n = 12).

10.5334/ijic.6905.s2 Appendix 2. Client and professional questionnaire about users’ experiences.

10.5334/ijic.6905.s3 Appendix 3. Codetree.

## Abbreviations

CJG: Centra for Youth and Family

EPR-Youth: Electronic Patient Record for Care for Youth

## Author Information

Janine Benjamins is a medical specialist in Public Health, working in the field of Preventive Child Healthcare since 1998. In the last decade she has been working as a policy advisor, co-designing innovations in ‘care for youth’ in her region, e.g. doctor-nurse task delegation in preventive child healthcare, transformation of care for youth towards client-centered care, and development of EPR-Youth. In feb 2019 she started as an external PhD-student at Wageningen University to investigate the effects of the use of EPR-Youth on interdisciplinary collaboration, clients’ autonomy and experienced quality of care.
