# Supplementary material for: Implementation of EPR-Youth, a Client-Accessible and Multidisciplinary Health Record; A Mixed-Methods Process Evaluation
Source: Int J Integr Care. 2023 Jun 16;23(2):26. doi: 10.5334/ijic.6905 (PMC10275189; doi:10.5334/ijic.6905)
Supplement: Appendix 1. — Overview of characteristics of focus group participants, shown separately for steering committee (n = 6), project group (n = 8), clients (n = 12), and professional users (n = 12). [file ijic-23-2-6905-s1.pdf]

Appendix 1: Overview of characteristics of focus group participants, shown separately for steering committee (n=6), project group (n=8), clients (n=12), and professional users (n=12).

	**Steering committee (all members)**	**n=6**
**Sex**	Male	3
	Female	3
**Role**	Manager	3
	Staff member	2
	Project leader	1
**Organization**	Youth care	1
	PCH 0-3	2
	PCH 4-18	2
	Other organization	1
**Setting**	Focus group	5
	Individual interview	1
	**Project group**	**n=8**
**Sex**	Male	2
	Female	6
**Profession**	Doctor	1
	Nurse	2
	Behavioural scientist	2
	Youth worker	1
	Administrative	1
	Application manager	1
**Organisation**	Youth care	3
	PCH 0-3	3
	PCH 4-18	2
	**Clients**	**n=12**
**Sex**	Male	4
	Female	8
**Parent or adolescent**	Parent	8
	Adolescent	4
**Educational level**	High	4
	Middle	5
	Low	3
**Native country**	the Netherlands	12
	Other	0
**PCH/ Youth care**	PCH	4
	Youth care	8
	**Professionals**	**n=12**
**Sex**	Male	1
	Female	11
**Profession**	Doctor	2
	Nurse	3
	Behavioral scientist	1
	Youth worker	3
	Administrative	2
	Screener	1
**Working experience**	Less than 5 years	4
	5 to 10 years	2
	More than 10 years	6
**Organization**	Youth care	4
	PCH 0-3	6
	PCH 4-18	2

PCH = preventive child healthcare
PCH 0-3 = preventive child healthcare for children up to 3 years old
PCH 4-18 = preventive child healthcare for children aged 4 to 18 years old.
