# Supplementary material for: Implementation of EPR-Youth, a Client-Accessible and Multidisciplinary Health Record; A Mixed-Methods Process Evaluation
Source: Int J Integr Care. 2023 Jun 16;23(2):26. doi: 10.5334/ijic.6905 (PMC10275189; doi:10.5334/ijic.6905)
Supplement: Appendix 2. — Client and professional questionnaire about users’ experiences. [file ijic-23-2-6905-s2.pdf]

Appendix 2: Client and professional questionnaire about users' experiences

Clients:
(Represented here is the version for parents. In the questionnaire among adolescents, the text 'your child's EPR-Youth' is replaced by 'your EPR-Youth')

1. Do you appreciate the possibility to read and write in your child's EPR?
    a. I appreciate that very much
    b. I appreciate that a little bit
    c. I neither appreciate nor dislike that
    d. I dislike that a little bit
    e. I dislike that very much
2. Did you ever log on to your child's record?
    a. No, never
    b. No, I tried but did not manage to get in
    c. Yes, I logged on to my child's record
3. What was the reason that you logged on to your child's record?
    a. I wanted to reread what we had discussed
    b. I wanted to check or change my appointment, or plan a new appointment
    c. I wanted to ask the CJG-professionals a question
    d. I wanted to add information (e.g., a plan)
    e. I wanted to do something else……
4. We want you to log on easily to your child's EPR-Youth. Do you manage to do that? *
    a. I manage easily
    b. I manage okay
    c. Neutral
    d. I manage with difficulty
    e. I never managed
5. We want you to understand what you read in your child's EPR-Youth. Do you? *
    a. I always do
    b. I do, most of the time
    c. I do, as often as not
    d. Sometimes, I do
    e. I never do
6. We want EPR-Youth to give you a clear overview of your child's situation and care plan. Does it? *
    a. Always
    b. Most of the time
    c. Just as often as not
    d. Sometimes
    e. Never

**: These questions use a 5-point Likers scale, ranging from one, totally agree, to five, totally disagree. (or an equivalent of these answers, if that matches the formulation of the question better).*

Professionals:
(Questions 1-13 use a 5-point Likers scale, ranging from one,' totally agree', to five, 'totally disagree'.)

1. I find EPR-Youth unnecessarily complex. **
2. I think that EPR-Youth is easy to use.
3. I think I will need the support of a technical person to be able to use EPR-Youth **.
4. I find that the various functions in EPR-Youth are well integrated.
5. I think there is too much inconsistency in EPR-Youth.**
6. I can imagine that most people will learn to use EPR-Youth very quickly.
7. I find EPR-Youth very cumbersome to use.**
8. I feel very confident using EPR-Youth.
9. I needed to learn a lot of things before I could get going with EPR-Youth**.
10. EPR-Youth offers everything I need to do my job.
11. EPR-Youth enables me to complete certain tasks faster than the previous system.

12. EPR-Youth makes administrative tasks take less time-consuming than the previous system.
13. Using EPR-Youth enhances the quality of my work, compared with the previous system.

***: These questions were reverse keyed. The response category 'agree' represents a negative opinion on this aspect of ease-of-use of EPR-Youth.*
