# Supplementary figures and images for: Implementation of EPR-Youth, a Client-Accessible and Multidisciplinary Health Record; A Mixed-Methods Process Evaluation
Source: Int J Integr Care. 2023 Jun 16;23(2):26. doi: 10.5334/ijic.6905 (PMC10275189; doi:10.5334/ijic.6905)

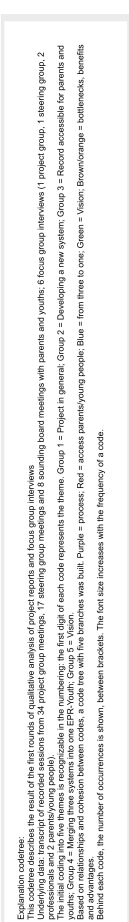[illegible]

Supplement: Appendix 3. — Codetree. [file ijic-23-2-6905-s3.pdf]
